# Left Gastric Vein Width Is an Important Risk Factor for Exacerbation of Esophageal Varices Post Balloon-Occluded Retrograde Transvenous Obliteration for Gastric Varices in Cirrhotic Patients

**DOI:** 10.3390/medicina58020205

**Published:** 2022-01-28

**Authors:** Taku Mizutani, Kazushige Nirei, Tatsuo Kanda, Masayuki Honda, Tomotaka Ishii, Shuhei Arima, Yoichiro Yamana, Naoki Matsumoto, Shunichi Matsuoka, Mitsuhiko Moriyama

**Affiliations:** Division of Gastroenterology and Hepatology, Department of Medicine, Nihon University School of Medicine, 30-1 Oyaguchi-kamicho, Itabashi-ku, Tokyo 173-8610, Japan; mizutani.taku@nihon-u.ac.jp (T.M.); kanda.tatsuo@nihon-u.ac.jp (T.K.); honda.masayuki@nihon-u.ac.jp (M.H.); ishii.tomotaka@nihon-u.ac.jp (T.I.); arima.shuhei73@nihon-u.ac.jp (S.A.); yamana.yoichiro@nihon-u.ac.jp (Y.Y.); matsumoto.naoki@nihon-u.ac.jp (N.M.); s.matsuoka@mitsuwadaibyoin.or.jp (S.M.); moriyama.mitsuhiko@nihon-u.ac.jp (M.M.)

**Keywords:** balloon-occluded retrograde transvenous obliteration, gastric varices, esophageal varices, left gastric vein

## Abstract

*Background and Objectives*: Balloon-occluded retrograde transvenous obliteration (BRTO) could be currently one of the best therapies for patients with gastric varices. This study examined the exacerbation rates for esophageal varices following BRTO for gastric varices in patients with hepatic cirrhosis. *Materials and Methods*: We enrolled 91 cirrhotic patients who underwent BRTO for gastric varices. In total, 50 patients were examined for exacerbation rates of esophageal varices following BRTO. Esophageal varices and their associated exacerbation were evaluated by upper gastrointestinal endoscopy. Patients were allocated into two groups according to the main inflow tract for gastric varices: (1) 37 patients in the left gastric vein (LGV) group with an LGV width of more than 3.55 mm, and (2) 13 patients in the non-LGV group who had short gastric vein or posterior gastric vein. Moreover, treatment outcomes were retrospectively analyzed. *Results*: LGV width (*p* < 0.01) was the major risk factor for the deterioration of esophageal varices post BRTO. In addition, LGV was the most common inflow tract, and the LGV group contained 74% (37/50) of patients. The exacerbation rates of esophageal varices at 1, 2, 3, and 4 years post BRTO were 40%, 62%, 65%, and 68%, respectively. The comparison of the exacerbation rates for esophageal varices following BRTO according to inflow tract showed that the exacerbation rates were significantly higher in the LGV group than those of the non-LGV group (*p* = 0.03). In more than half of the subjects, LGV was the main inflow tract for gastric varices, and this group experienced more frequent exacerbations of esophageal varices following BRTO compared to patients with different inflow tract sources. *Conclusion*: Careful attention should be paid to the LGV width when BRTO is performed for gastric varices.

## 1. Introduction

Gastric varices are serious complications that result from portal hypertension in patients with or without cirrhosis [1]. In general, it may be difficult to control extensive bleeding from gastric varices [2]. Most gastric fundic varices formed by large spontaneous shunts in either gastric or splenic veins are continuous with the left renal vein via the suprarenal (adrenal) vein [3,4]. Thus, gastric varices which are associated with porto-systemic shunt (PSS)—including gastro-renal shunt—have a five-year cumulative bleeding rate of 44% if left untreated, with a much lower survival rate associated with rebleeding [5].

Endoscopic sclerotherapy, transjugular intrahepatic portosystemic shunt (TIPS), and balloon-occluded retrograde transvenous obliteration (BRTO) [6,7,8,9,10] are generally used for the management of gastric variceal bleeding. BRTO is a reasonable treatment for gastric varices because the outflow route from most gastric varices connects to the left renal vein [1]. A meta-analysis demonstrated that BRTO is a safe and efficacious treatment for gastric varices and BRTO could be currently one of the best therapies for patients with gastric varices [11]. BRTO is an established treatment for solitary gastric varices and hepatic encephalopathy due to PSS and is widely applied in Japan [6,7] since the Japanese health insurance system has approved endoscopic sclerotherapy and BRTO as treatment options for the gastric varices.

There is a well-known correlation between the size and course of the left gastric vein (LGV) and esophageal varices [12]. Jogo et al. reported on the factors associated with aggravation of esophageal varices after performing BRTO for gastric varices [13]. They showed that total bilirubin and hepatic vein pressure gradients are important independent risk factors for worsening esophageal varices post BRTO [13]. Maruyama et al. reported the association between LGV width and esophageal varices with an optimal cutoff value of the LGV diameter—to identify any esophageal varices—of 3.55 mm [14].

Here, we retrospectively analyzed the responsible vessels of gastric varices using three-dimensional-computed tomography (3D-CT) and compared exacerbation rates of esophageal varices among patients with successful BRTO.

## 2. Materials and Methods

### 2.1. Patients

**Figure 1:**
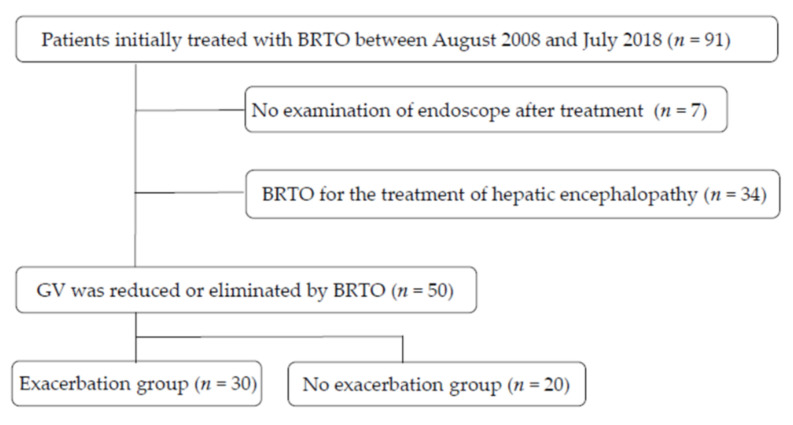
Flowchart showing patients enrolled in this study. BRTO: balloon-occluded retrograde transvenous obliteration; GV: gastric varices.

A total of 91 patients treated with BRTO (between 2008 and 2018) for solitary gastric varices were initially included. Seven patients who were transferred to another hospital shortly after receiving BRTO and who did not receive an endoscopic examination were excluded. A total of 34 patients who received shunt embolization for the treatment of hepatic encephalopathy were also excluded from this study. We retrospectively analyzed their data from a total of 50 cirrhotic patients (Figure 1). This study was approved by the Hospital Institutional Review Board of the Nihon University School of Medicine Itabashi (RK-200714-7) and conformed to the ethical guidelines of the Declaration of Helsinki. Participation in the study was posted on the institution’s website, and informed consent was obtained from all patients.

### 2.2. Eligibility Criteria of the BRTO Treatment for Gastric Varices

Eligibility criteria of gastric varices treatment were defined based on the Code for the Management of Portal Hypertension, Revised Third Edition, 2013, issued by the Japan Society for Portal Hypertension [15]. The criteria included the following: red color sign (RCS)-positive, erosion or ulcer formation on varices, engorgement classified as form F2 to F3, tendency toward a rapid short-term increase, and residual or novel gastric varices after treatment of esophageal varices [15]. In addition, gastro-renal shunts, which were likely to be embolized, were confirmed. The status of gastric varices post BRTO was recorded as either improvement (form 1 or more) or disappearance and esophageal varices aggravation (form 1 or more), according to guidelines stipulated by the Japan Society for Endoscopic Surgery. For evaluation of esophageal varices before and post BRTO, a patient with a score that worsened or a patient who became form 1 or worse was defined as “exacerbation” (e.g., F0 to F1, F2 or F3; F1 to F2 or F3) [15]. After treatment of gastric varices by BRTO, patients whose esophageal varices worsened within 1 year or patients without exacerbation of esophageal varices were defined as the (esophageal varices) exacerbation group or as the non-exacerbation group, respectively.

### 2.3. Endoscopic Grading of Esophageal, Endoscopic Examination and Treatment

Endoscopic grading of esophageal varices was defined according to the guidelines of the Japanese Research Society for Portal Hypertension [15]. Esophageal varices were classified as follows: F1—small straight varix; F2—enlarged tortuous varix occupying less than one-third of the lumen; F3—large coil-shaped varix occupying more than one-third of the lumen.

In general, we performed endoscopy within 1 to 6 months after performing BRTO. Then, endoscopy was performed every 3 to 6 months. At our hospital, endoscopic treatment is performed in patients with esophageal varices F2 or more, and in RCS-positive patients who undergo endoscopic follow-up post BRTO. Three-dimensional-computed tomography (3D-CT) was used to confirm the inflow tract for gastric varices prior to BRTO in all 50 patients (Figure 1).

### 2.4. Performing Three Dimensional-Computed Tomography (3D-CT)

**Figure 2:**
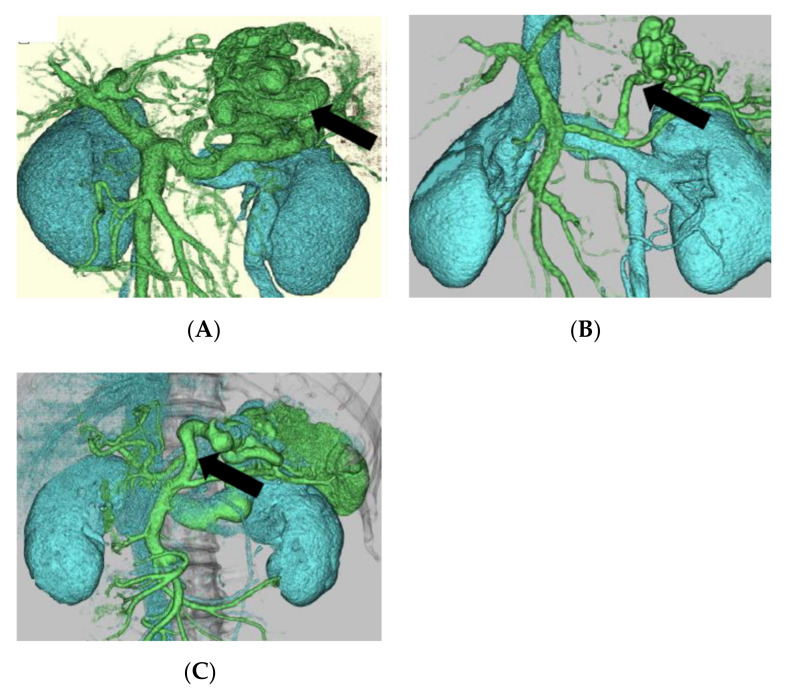
Inflow tract for gastric varices assessed by three-dimensional-computed tomography (3D-CT). Inflow tract for gastric varices before performing balloon-occluded retrograde transvenous obliteration (BRTO) was confirmed by 3D-CT. Inflow tract for gastric varices was from (**A**) short gastric vein (SGV) (non-LGV group), (**B**) SGV and posterior gastric vein (PGV) (non-LGV group), or (**C**) left gastric vein (LGV group).

Prior to treatment, patients underwent plain CT and enhanced 3D-CT. Inflow tract for gastric varices was assessed by enhanced 3D-CT. The patients were divided into two groups: (1) LGV group and (2) non-LGV group. Patients with an LGV width of more than 3.55 mm were defined as the LGV group, and LGV was the main inflow tract [14]. The non-LGV group consisted of patients in which the inflow tract was from the short gastric vein (SGV) (Figure 2A) or posterior gastric vein (PGV) (Figure 2B) rather than the LGV (Figure 2C). The non-LGV group included patients with an LGV diameter of 3.55 mm or less [14].

### 2.5. Procedure of Balloon-Occluded Retrograde Transvenous Obliteration (BRTO)

An 8 Fr-long guiding sheath (ASATO; MEDIKIT, Tokyo, Japan) was inserted from the right femoral vein into the left renal vein via the inferior vena cava (IVC) using a guidewire (PIOLAX Hydrophilic Guidewire; SURF, Yokohama, Japan). We performed BRTO using a 5 Fr guiding balloon catheter (CANDIS; MEDIKIT) or Selecon MP catheter (TERUMO, Tokyo, Japan) equipped with a balloon catheter. For the BRTO procedure, a hardening agent (5% ethanolamine oleate with iopamidol (EOI)) was infused under balloon occlusion, and a balloon catheter was placed for at least six hours. For patients who were considered to have higher blood flow, the catheter was placed overnight and haptoglobin was administered the day before and on BRTO treatment to prevent hemolysis due to the hardening agent. After treatment, antibiotics were prophylactically used to prevent infection. Complete blockage of the shunt was confirmed by 3D-CT.

### 2.6. Laboratory Tests

Laboratory tests were performed at least every 4 weeks before and after the BRTO procedure. Prior to starting BRTO, we measured the levels of serum aspartate aminotransferase (AST), alanine aminotransferase (ALT), total bilirubin, creatinine, prothrombin time, total cholesterol, albumin levels, platelet counts, hemoglobin, and white blood cells (WBC). Hepatitis B virus (HBV) surface antigen (HBsAg) and anti-hepatitis C virus (HCV) antibodies were also measured in all patients. Diagnosis of cirrhosis and/or hepatocellular carcinoma was previously described [16].

### 2.7. Statistical Analysis

Statistical analyses were performed in order to determine the exacerbation rates of esophageal varices following BRTO in all patients and to compare the exacerbation rates of esophageal varices following BRTO between the LGV group and the non-LGV group. Differences between the LGV group and the non-LGV group were analyzed using the Mann–Whitney *U*-test, chi-squared test, and Wilcoxon signed-rank test.

The cumulative incidence of esophageal varices between both groups was compared using Gray’s test. All statistical analyses were performed using EZR (Easy R) software, a modified version of R commander designed to add statistical functions frequently used in biostatistics. EZR is freely available at (http://www.jichi.ac.jp/saitama-sct/SaitamaHP.files/statmed.html, accessed on 1 December 2021), which is a modified version of R commander, designed to add frequently used statistical functions in biostatistics [16,17].

## 3. Results

### 3.1. Effects and Complications of Balloon-Occluded Retrograde Transvenous Obliteration (BRTO)

In all 50 patients, gastric varices were reduced or eliminated by BRTO. Complete blockage of the shunt was confirmed by 3D-CT within three weeks post BRTO. Although the timing of gastric varices varied among these subjects, no morphological exacerbation of gastric varices and bleeding were observed. Complications due to BRTO consisted of hemolysis caused by the hardening agent, which was observed in 4 out of 50 patients (8%); they all improved spontaneously within 24 h. Although 7 out of 50 patients (14%) experienced fever over 38 °C, all patients improved within two days. There were no serious complications (i.e., those caused by the catheterization, pulmonary embolism, bleeding, renal failure, and hepatic failure). Overall, this procedure is generally considered as safe.

### 3.2. Comparison of Pretreatment Factors between Esophageal Varices-Exacerbation Group and Non-Exacerbation Group

Table 1 shows the background features of patients between the esophageal varices exacerbation group and non-exacerbation group. Results from the univariate analysis demonstrated higher hemoglobin levels (*p* = 0.01) and platelet counts that tended to be lower (*p* = 0.07) in the esophageal varices exacerbation group. Moreover, we observed a higher proportion of patients with an LGV width of more than 3.55 mm in the esophageal varices-exacerbation group than in the non-exacerbation group (*p* = 0.01).

### 3.3. Comparison of Pretreatment Factors between LGV Group or Non-LGV Group Patients

Next, we compared the background features between the LGV group and the non-LGV group (Table 2). The LGV group showed a lower platelet count, higher AST levels, higher ALT levels, and HCV etiology, compared to the non-HCV group (Table 2).

The LGV group included 37 patients (74%), while the non-LGV group included 13 patients (26%). The mean follow-up periods of the LGV group and non-LGV group were 37.73 ± 28.83 and 29.14 ± 26.67 months, respectively (*p* = 0.32). Prior to BRTO treatment, the prevalence of gastric varices or esophageal varices was not statistically significant between both groups (Table 2). Exacerbation of esophagus varices post-BRTO treatment was observed to a greater extent in the LGV group than in the non-LGV group (*p* = 0.02). The mean period from BRTO to esophageal varices exacerbation tended to be shorter in the LGV group than in the non-LGV group (*p* = 0.06) (Table 2).

### 3.4. Esophageal Varices were Significantly Exacerbated Post-Balloon-Occluded Retrograde Transvenous Obliteration (BRTO) in the Left Gastric Vein Group (LGV Group)

**Figure 3:**
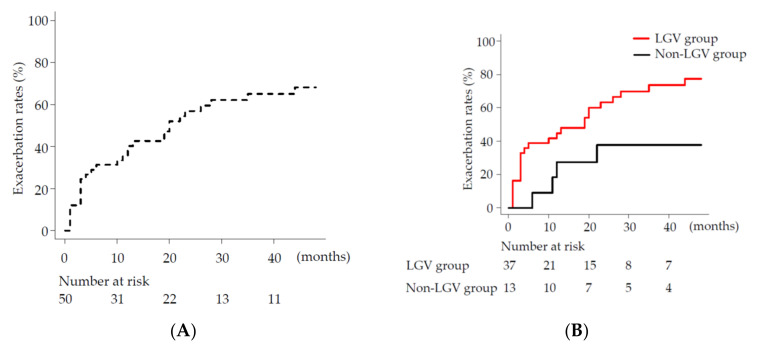
Exacerbation rates (%) of esophageal varices following balloon-occluded retrograde transvenous obliteration (BRTO). (**A**) Overall exacerbation rates (%) (dotted line); (**B**) exacerbation rates (%) of left gastric vein (LGV) group (red line) and non-LGV group (black line).

The overall exacerbation rates of esophageal varices post BRTO were: 40%, 62%, 65%, and 68% at 1, 2, 3, and 4 years, respectively (Figure 3A). Next, the exacerbation rates of esophageal varices—following BRTO according to the inflow tract of gastric varices—were examined and compared (Figure 3B). The exacerbation rates of esophageal varices at 1, 2, 3 and 4 years post BRTO were: 48%, 63%, 73% and 74%, respectively, in the LGV group; in the non-LGV group—18%, 37%, 37% and 37%. Comparing both the LGV and non-LGV groups, esophageal varices were significantly exacerbated in the LGV group (Gray test: *p* < 0.03).

## 4. Discussion

Normal blood flow in the portal vein is antegrade and hepatopetal. However, blood vessels that form the portal venous system may become partially retrograde and show hepatofugal flow when the portal blood pressure elevates [18]. Once cirrhosis is established, collateral circulation is primarily formed as esophageal varices and gastric varices. Ruptured esophageal varices and gastric varices occasionally cause death from bleeding or hepatic failure; they are also serious complications affecting the prognosis of patients with cirrhosis. In particular, ruptured gastric varices in patients with gastro-renal shunts cause increased blood inflow and larger hemorrhages [9,19]. The cumulative incidence rates of bleeding from fundal varices within 1, 3, and 5 years have been reported as 16%, 36%, and 44%, respectively [5]. The need for prophylactic treatment of gastric varices at risk of rupture has been recognized [20,21], and BRTO is widely used in Japan as first-line therapy for fundal varices [22,23,24]. According to a report regarding the efficacy of BRTO with respect to bleeding, the cumulative incidence rates of bleeding within 1, 3, and 5 years were 0%, 0%, and 17%, respectively, in patients who received BRTO, and 19%, 41%, and 61%, respectively, in patients who did not [25]. Similarly, in our case, no bleeding was observed in patients. However, in the LGV group—BRTO treatment of gastric varices—esophageal varices occurred at a high rate of 74% in 4 years (Figure 3B). An LGV width of more than 3.55 mm appeared as an associated factor of exacerbation of esophageal varices.

Thus, the efficacy of BRTO has already been demonstrated. BRTO mainly interrupts the blood flow of gastro-renal shunt with a balloon and retrograde infusion of 5% EOI. The vascular endothelial cell membrane is impaired directly by ethanolamine oleate (EO), and fibrin and blood platelets attach to the endodermis, forming a thrombus [26]. This thrombus produces embolic effects and blocks the gastro-renal shunt blood flow of the gastric varices, which leads to an improvement and the elimination of gastric varices. Early thrombogenesis causes reduced blood flow, promoting morphological changes and collapse of the gastric varices, in which, several weeks later, a reduction in the organized thrombus occurs. Thus, the therapeutic effects are mainly monitored by 3D-CT in the early stage. It was reported that, in most cases, thrombogenesis occurs a week after, as assessed by 3D-CT [27]. In comparison, elimination of gastric varices and gastro-renal shunt has also been reported as taking approximately 1–3 months, and in certain patients, a larger shunt diameter requires a longer time for blockage of the blood flow [28,29]. We also performed this method and obtained good results.

In patients successfully treated by BRTO, thrombus-mediated gastro-renal shunt occlusion was reported to effectively improve liver function by increasing blood flow through the hepatic portal vein [30,31]. However, because the portal blood pressure exiting the gastro-renal shunt increases gradually after treatment, patients often experience novel or aggravated collateral circulation, particularly esophageal varices. In general, the reported cumulative incidence of exacerbation of esophageal varices post BRTO for gastric varices ranges from 10–63% [32,33]. Our results showed similar rates (65% at three years; 68% at four years) to those reported in [32,33]. Many studies on inflow tract for gastric varices reported that gastric varices blood flow is mainly supplied by the SGV and PGV [3]. Hirota et al. described that, in general, the recurrence of esophageal varices post BRTO for grades 1 and 2 (according to Hirota’s classification) is relatively low [34]. However, in our analysis, the exacerbation rates of esophageal varices following BRTO were significantly higher in the LGV group compared to those in the non-LGV group. Of note, there were no significant differences in the Child-Pugh grade between the LGV group and the non-LGV group. Therefore, further studies for portal hypertension—which seemed to be associated with exacerbation of esophageal varices, ascites, hepatic encephalopathy—are needed. Thus, we judged that it was the difference in the inflow tract for gastric varices that was associated with the exacerbation of esophageal varices post BRTO. Most inflow tracts forming esophageal varices are from the LGV. Blood flow upward from the LGV to the esophagus is increased by blocking blood flow of the gastro-renal shunt with BRTO, which seems to contribute to the exacerbation of esophageal varices. This is further supported by a report demonstrating an increase in LGV pressure and blood flow rate post BRTO using an ultrasonic autoscope [35].

Choe et al. conducted a retrospective analysis of patients with gastric varices and cirrhosis who underwent either endoscopic varicose vein occlusion (EVO) or BRTO as prophylactic treatment was observed without procedural intervention. After 35 months of observation, patients who underwent EVO or BRTO reported significantly less bleeding from gastric varices than patients with follow-up alone. Importantly, EVO and BRTO are effective and safe first-line preventive treatments that prevent bleeding from gastric varices. In particular, BRTO is superior to EVO in the complete eradication of gastric varices [36].

Furthermore, it has been noted that additional partial splenic embolization (PSE) post BRTO may reduce the incidence of esophageal varices relative to BRTO alone [37]. A previous report stated that the cumulative incidence rates of RCS-positive esophageal varices at 6 months, 1 year and 2 years were 16%, 27%, and 45%, respectively, in patients treated with BRTO alone, and 0%, 0%, and 9%, respectively, in patients treated with BRTO plus PSE [38]. Oshita et al. reported that splenectomy with gastric devascularization resulted in more effective liver function improvement than BRTO [39]. Thus, the therapeutic combination of BRTO with PSE should be considered for patients with LGV as the main inflow tract for gastric varices. We did not treat with PSE. However, if this were the case, it was theorized that the results of exacerbation of esophageal varices could be improved.

In addition, Jang et al. reported 183 cirrhotic patients who underwent BRTO for gastric varices bleeding. In their study, 52.3% of patients treated for gastric varices bleeding achieved eradication of gastric varices bleeding, along with a 72.8% reduction in gastric varices to grade 0 or 1. Postoperatively, esophageal varices occurred in 41.2% [40]. Moreover, patients who have undergone BRTO may require regular endoscopy for follow-up of esophageal varices, with or without treatment. In our study, 68% of esophageal varices worsened in 4 years. Therefore, attention should be given to LGV as an inflow tract and LGV width.

We performed the follow-up CT in 49 of 50 patients treated with BRTO and 3D-CT in 31 of these 49 patients after the initial 3D-CT following BRTO. The development of repermeabilized vein after BRTO and other collaterals than LGV, respectively, were observed in 3 and 4 of them (Table 3). Only one patient had both the development of repermeabilized vein after BRTO and inferior mesenteric vein collaterals.

## 5. Conclusions

Attention should be paid to the width of LGV, which is one of the inflow tracts for gastric varices, when BRTO is performed for gastric varices.

## Figures and Tables

**Table 1:** Comparison of patients’ background between the esophageal varices exacerbation and non-exacerbation groups.

	Total	Exacerbation Group	Non-Exacerbation Group	* *p*-Value
Number of patients	50	30	20	
Age (years)	67.38 ± 9.55	67.63 ± 9.47	67.00 ± 9.91	0.93
Gender (male/female)	32/18	20/10	12/8	0.63
WBC (×10/mm^3^)	6.97 ± 0.63	4.40 ± 1.62	4.81 ± 2.69	0.86
Hemoglobin (g/dL)	12.09 ± 54.44	13.16 ± 6.63	10.48 ± 2.20	0.01
Platelet counts (×10^4^/mm^3^)	9.77 ± 4.22	8.63 ± 2.98	11.47 ± 5.23	0.07
AST (IU/L)	58.50 ± 39.16	59.96 ± 41.40	56.30 ± 36.48	0.86
ALT (IU/L)	42.40 ± 34.29	44.90 ± 34.61	38.65 ± 34.35	0.33
Total bilirubin (mg/dL)	1.17 ± 0.76	1.19 ± 0.58	1.13 ± 0.58	0.22
Total protein (g/dL)	6.96 ± 0.63	7.05 ± 0.62	6.85 ± 0.65	0.32
Albumin (g/dL)	3.42 ± 0.71	3.45 ± 0.82	3.36 ± 0.49	0.96
Creatinine (mg/dL)	0.71 ± 0.20	0.73 ± 0.17	0.68 ± 0.24	0.18
eGFR (mL/min/1.73 m^2^)	80.48 ± 22.85	76.26 ± 18.66	87.52 ± 27.67	0.15
Prothrombin time (%)	83.42 ± 13.83	84.83 ± 12.57	81.30 ± 15.62	0.52
Prothrombin time (INR)	1.13 ± 0.15	1.12 ± 0.14	1.15 ± 0.16	0.64
Total cholesterol (IU/L)	148.31 ± 37.23	145.07 ± 37.01	152.85 ± 38.01	0.77
Blood glucose (mg/dL)	130.52 ± 43.12	132.0 ± 48.96	128.3 ± 33.59	0.87
NH_3_ (μg/dL)	75.04 ± 35.48	72.25 ± 35.23	79.15 ± 3690	0.52
Child-Pugh A/B/C	39/11/0	24/6/0	15/5/0	0.73
HCC (±)	27/23	17/13	10/10	0.77
GV form F1/2/3	2/17/31	0/11/19	2/6/12	0.60
EV form F0/1/2/3	30/17/2/1	19/9/2/0	11/8/01	0.62
Etiology (HBV/HCV/NBNC/Alcohol)	3/28/7/12	2/20/3/5	1/8/4/7	0.27
LGV > 3.55 mm (yes/no)	37/13	26/4	11/9	0.01
Mean observation periods (months)	35.49 ± 28.28	32.73 ± 28.13	39.63 ± 28.71	0.42

Data are expressed as the mean ± standard deviation. * *p*-Value, comparison between two groups, by univariate analysis; WBC—white blood cell counts; AST—aspartate aminotransferase; ALT—alanine aminotransferase; eGFR—estimated glomerular filtration rate; HCC—hepatocellular carcinoma; GV—gastric varices; EV—esophageal varices; HBV—hepatitis B virus; HCV—hepatitis C virus; NBNC—non-HBV, non-HCV; LGV—left gastric vein.

**Table 2:** Comparison of patients’ background between left gastric vein (LGV) and non-LGV groups.

□	LGV Group	Non-LGV Group	* *p*-Value
Number	37	13	□
Age (years)	66.8 ± 10.2	68.8 ± 7.5	0.69
Gender (male/female)	12/25	6/7	0.50
WBC (×10/mm^3^)	467 ± 232	426 ± 132	0.98
Hemoglobin (g/dL)	12.4 ± 6.2	11.2 ± 2.0	0.53
Platelet counts (×10^4^/mm^3^)	9.1 ± 3.7	11.6 ± 5.1	0.13
AST (IU/L)	63.8 ± 43.6	43.4 ± 14.5	0.17
ALT (IU/L)	46.9 ± 38.6	29.4 ± 38.56	0.11
Total bilirubin (mg/dL)	1.24 ± 0.85	0.95 ± 0.30	0.41
Total protein (g/dL)	7.00 ± 0.58	6.88 ± 0.77	0.65
Albumin (g/dL)	3.40 ± 0.76	3.48 ± 0.56	0.30
Creatinine (mg/dL)	0.7 ± 0.2	0.6 ± 0.2	0.80
eGFR (mL/min/1.73 m^2^)	80.1 ± 22.8	81.5 ± 23.9	0.89
Prothrombin time (%)	83.0 ± 13.8	84.3 ± 14.2	0.64
Total cholesterol (IU/L)	151.4 ± 39.8	138.8 ± 27.2	0.21
Blood glucose (mg/dL)	131.9 ± 46.0	126.4 ± 34.8	0.82
NH_3_ (μg/dL)	75.3 ± 34.0	74.3 ± 41.0	0.66
Child-Pugh A/B/C	27/10/0	12/1/0	0.24
HCC (±)	20/17	7/6	1.0
GV form F1/2/3	1/12/24	1/5/7	0.62
EV form F0/1/2/3	23/11/2/1	7/6/0/0	0.72
Etiology (HBV/HCV/NBNC/Alcohol)	1/22/4/10	2/6/3/2	0.17
Esophagus varices exacerbation	26/37	4/13	0.02
Mean periods from BRTO to esophageal varices exacerbation (months)	17.6 ± 17.0	27.2 ± 17.3	0.06
Mean observation periods (months)	37.73 ± 28.83	29.14 ± 26.67	0.32

Data are expressed as the mean ± standard deviation. * *p*-value, comparison between two groups, by univariate analysis; WBC—white blood cell counts; AST—aspartate aminotransferase; ALT—alanine aminotransferase; eGFR—estimated glomerular filtration rate; HCC—hepatocellular carcinoma; GV—gastric varices; EV—esophageal varices; HBV—hepatitis B virus; HCV—hepatitis C virus; NBNC—non-HBV, non-HCV; LGV—left gastric vein.

**Table 3:** Six patients with the development of repermeabilized vein, other collaterals other than left gastric vein, following balloon-occluded retrograde transvenous obliteration.

**Case**	**Age (years)/** **Gender**	**Hemoglobin (g/dL)**	**Platelet Counts (×10^4^/mm^3^)**	**AST (IU/L)**	**ALT (IU/L)**		**Creatinine (mg/dL)**	**Child-Pugh A/B/C (Score)**	**HCC (±)**
1	53/male	5.7	8	37	40		0.79	B-7	+
2	63/male	13.6	6.9	57	45		0.49	A-6	+
3	64/female	9.3	10.6	31	24		0.7	A-5	+
4	51/male	13.8	8.1	51	27		0.67	A-6	−
5	63/female	8.8	10.5	56	36		0.35	A-5	+
6	78/male	14	16.5	71	57		1.12	A-6	−
**Case**	**GV Form**	**EV Form**	**Etiology**	**LGV > 3.55 mm**	**LGV Group (yes/no)**	**Esophageal Varices** **Exacerbation (yes/no)**	**Repermeabilized** **Vein (±)**	**Other Collaterals than LGV**	**Occurrence** **Following BRTO (months)**
1	F3	F1	HBV	5.68	Yes	No	+	-	9
2	F2	F2	HCV	5.45	Yes	Yes	+	-	26
3	F2	F1	HCV	2.56	No	Yes	+	Inferior mesenteric vein	8
4	F2	F1	NBNC	2.55	No	Yes	-	Spleno-renal shunt	5
5	F3	F1	NBNC	3.34	No	No	-	Abdominal wall veins	24
6	F2	F0	Alcohol	5.61	Yes	No	-	Paraumbilical vein	36

AST—aspartate aminotransferase; ALT—alanine aminotransferase; HCC—hepatocellular carcinoma; GV—gastric varices; EV—esophageal varices; HBV—hepatitis B virus; HCV—hepatitis C virus; NBNC—non-HBV, non-HCV; LGV—left gastric vein.

## Data Availability

All the data underlying this article are available in this article.
